# Decoding Ecuadorian *Mycobacterium tuberculosis* Isolates: Unveiling Lineage-Associated Signatures in Beta-Lactamase Resistance via Pangenome Analysis

**DOI:** 10.3390/biomedicines13020313

**Published:** 2025-01-28

**Authors:** Gabriel Morey-León, Juan Carlos Fernández-Cadena, Derly Andrade-Molina, Luisa Berná

**Affiliations:** 1 Facultad de Ciencias de la Salud, Universidad Espíritu Santo, Samborondón 0901952, Ecuador; 2 Facultad de Ciencias, Universidad de la República, Montevideo 11400, Uruguay; 3 African Genome Center, University Mohammed VI Polytechnic (UM6P), Ben Guerir 43150, Morocco; juan.fernandez@um6p.ma; 4 Laboratorio de Ciencias Ómicas, Universidad Espíritu Santo, Samborondón 0901952, Ecuador; dmandrademolina@uees.edu.ec; 5 Laboratorio de Interacciones Hospedero-Patógeno, Unidad de Biología Molecular, Institut Pasteur de Montevideo, Montevideo 11400, Uruguay; 6 Unidad de Genómica Evolutiva, Facultad de Ciencias, Universidad de la República, Montevideo 11400, Uruguay; 7 Institut Pasteur de Montevideo, Montevideo 11400, Uruguay

**Keywords:** pangenome, core-genome, tuberculosis, beta-lactamase, Ecuador, surveillance

## Abstract

**Background:** Tuberculosis is the second largest public health threat caused by pathogens. Understanding *Mycobacterium tuberculosis*’s transmission, virulence, and resistance profile is crucial for outbreak control. This study aimed to investigate the pangenome composition of *Mycobacterium tuberculosis* clinical isolates classified as L4 derived from Ecuador. **Methods:** We analyzed 88 clinical isolates of *Mycobacterium tuberculosis* by whole-genome sequencing (WGS) and bioinformatic tools for Lineage, Drug-resistance and Pangenome analysis. **Results:** In our analysis, we identified the dominance of the LAM lineage (44.3%). The pangenomic analysis revealed a core genome of approximately 3200 genes and a pangenome that differed in accessory and unique genes. According to the COG database, metabolism-related genes were the most representative of all partitions. However, differences were found within all lineages analyzed in the metabolic pathways described by KEGG. Isolates from Ecuador showed variations in genomic regions associated with beta-lactamase susceptibility, potentially leading to epistatic resistance to other drugs commonly used in TB treatment, warranting further investigation. **Conclusions**: Our findings provide valuable insights into the genetic diversity of *Mycobacterium tuberculosis* populations in Ecuador. These insights may be associated with increasing adaptation within host heterogeneity, variable latency periods, and reduced host damage, collectively contributing to disease spread. The application of WGS is essential to elucidating the epidemiology of TB in the country

## 1. Introduction

Tuberculosis (TB) caused by *Mycobacterium tuberculosis* (*Mtb*) is a significant global health concern, with an estimated 10.8 million infections and 1.25 million deaths in 2023 [1]. *Mtb* has encompassed complex conformed of genetically related bacteria including *M. africanum* [2,3], which, within *Mtb*, are the typical human pathogens, and other lineages that infect animals (*M. bovis*, *M. canetti*, *M. microti*, *M. caprae*, *M. pinnipedii*, and *M. orygis*) [4,5,6,7], which have evolved from common ancestors over centuries from different geographic areas, and adapting to external conditions [8,9,10]. Some studies have demonstrated the relationship between clinical isolates and specific genotypes and geographical regions as well as their remarkable capacity for increased dissemination that sets them apart from ancestral lineages [11,12,13,14]. This is particularly evident due to their higher virulence and shorter latency periods to change the active form.

Furthermore, it has been noted that even within modern lineages, which are known for their heightened virulence and rapid dissemination, not all isolates display identical characteristics [15]. This diversity in behavior is influenced by various external factors, including drug resistance, host heterogeneity, demographic factors, and the presence of dominant lineages that confer advantages in terms of dissemination and impact on the host [10,16]. In Ecuador, Lineage Euro-American (L4) is the most predominant, and molecular epidemiology studies have shown that the population structure of *Mtb* is composed of LAM, X-type, and Haarlem sublineages [17,18,19,20,21].

Genomic approaches, such as WGS of *Mtb* strains, have improved the understanding of composition analysis and provided invaluable information on gain or loss genes, evolutionary markers, and polymorphisms related to drug resistance, virulence, and sub-lineage patterns [22,23,24,25,26,27,28]. With a large amount of information on genes from sequence isolates, the pangenome-based approach is more convenient for discerning a complete analysis in search of unique, accessory, and core genes, estimating the diversity of genes and novel marker genes, especially local-distribution-associated [29,30], mostly associated with virulence and drug resistance.

The pangenome represents the complete set of genes present in a species. It comprises the core genome, consisting of genes shared by all species members, the accessory genome, which includes genes present in some but not all members, and the unique genome, which includes genes present in only one species member [31]. Additionally, the cloud genome refers to a subset of genes in the accessory genome that are not universally distributed but may be present in certain subgroups or populations under specific environmental conditions or selective pressures [32]. Pangenome studies have been performed on different isolates from Peru [33], Mexico [34], Brazil, Argentina, Paraguay, and Colombia [35] from clinical samples of *Mtb* understand the variation in terms of unique sequences among them and to identify the importance of genes related to metabolism to adapt to external conditions and latency periods. However, no extensive pangenome studies have been performed in Ecuador to reveal the composition of the circulating lineages in the country. A unique WGS study identified the presence of 4.3.2/3 (LAM) and 4.1.2 (Haarlem) sub-lineages in a small dataset of *Mtb* strains from Ecuador [36]. In this study, we aimed to investigate the genomic composition of 88 *Mtb* strains classified as L4 derived from Ecuador using pangenome analysis. In addition, we explored the genetic variation in genomic regions associated with β-lactamase susceptibility using bioinformatics-based inference methods. Our findings provide valuable insights into the genetic diversity of *Mtb* in Ecuador and suggest an association of lineage with specific mutations related to β-lactamase resistance in TB, which drives the development of more effective TB control strategies tailored to the specific characteristics of the local *Mtb* population in the region.

## 2. Materials and Methods

### 2.1. Mtb Samples, Assembly, Functional Annotation, and Pangenome Analysis

This study included the raw reads from 88 clinical isolates of *Mtb* collected from different provinces in Ecuador between 2019 to 2021, which are available in the Sequence Read Archive (SRA) database (http://www.ncbi.nlm.nih.gov/sra, accessed on 15 June 2024) under the PRJNA827129 BioProject. Of the total isolates, 81.8% were from Guayaquil, while the remaining isolates were distributed as follows: Babahoyo (5.6%), El Empalme (3.4%), and Quito (2.3%). This distribution highlights the geographical spread and prevalence of TB in these areas. A small proportion of isolates came from Chone, Durán, Guaranda, Machala, and Nueva Loja, with each contributing 1.1% of the total. Raw reads were quality-checked using FastQC v0.11.9 [37] and improved by Trimmomatic v0.38 [38]. Specie confirmation and contamination screening was performed by Kraken v2 [39]. A Unicycler assembly pipeline [40] was used to assemble high-quality reads with a minimum contig size of 300 bp and polished discordance using Pilon v1.24 [41]. The quality of the assemblies was evaluated using *Mtb* strain HR37v (NC_000962.3) as a reference genome in Quast v5.0.2. [42]. Structural and functional annotation was performed using Rapid Annotation with the Subsystem Technology tool kit (RASTtk) in the Pathosystems Resource Integration Center (PATRIC) (https://www.patricbrc.org/, accessed on 1 July 2024). Pangenome analysis was performed using Roary v3.11.2 [43] and BPGA (Bacterial Pan-Genome Analysis tool) pipeline v1.3.0 [44]. Roary was performed with a minimum identity percentage for blastp of 95%, and gene detection in 99% of the isolates was recorded as a core gene. Roary_stats, a custom R tool v4.4.0, was used to process the outputs. In the BPGA pipeline, the COG and KEEG databases were used to define the functionality of the characterized gene. The COG database is embedded in the BPGA pipeline to facilitate the functional annotation of genes, helping researchers to understand the functional landscape of the bacterial genomes whilst the KEGG database is integrated into the BPGA pipeline to facilitate the annotation of genes involved in metabolic and signaling pathways. This helps researchers gain a deeper understanding of the biological processes, metabolic capabilities, and functional diversity of bacterial genomes in their pangenomic analysis. The pangenome was divided into three partitions: core, accessory, and cloud/unique. Core genes were present in 100% of the isolates, accessory in 3–99%, and cloud/unique in 1–2%.

### 2.2. Variant Calling, Lineage Classification, and Drug-Resistance Genes

Genetic relatedness among 88 *Mtb* genomes was processed using the MTBseq pipeline v1.1.0 with standard input parameters [45]. Briefly, BWA-mem and SAMtools algorithms were used to map the reads to the Mtb reference genome (NC_000962.3). GATK v3 was applied for base call recalibration and realignment of reads around InDels, and samtools mpileup for variant calling. Good-quality genomes presented a minimum mean coverage > 20Xx, read depth DP < 5, and reference genome covered >95%. The sub-lineage classification was performed using the strain module to perform lineage classification based on a set of phylogenetic SNPs, and a genetic distance matrix among transmission groups was created from the MTBseq result of the TBgroups module, which infers related isolates based on pairwise distance between distinct SNP positions. The TB-Profiler v4 pipeline was used to predict canonical mutations in the genes associated with resistance to first- and second-line drugs. We used BCFtool as the caller algorithm within the TB-profiler. For beta-lactamase susceptibility analysis, 46 genes were selected due to being especially related to beta-lactamase resistance and cell wall biosynthesis.

## 3. Results

### 3.1. Assembly, Functional Annotation, and Pangenome Analysis

In this study, we conducted a comparative genome analysis of 88 *Mtb* isolates from Ecuador. The results reveal an average genome size of 4.32 Mb (range: 4.21–4.33) and a high GC content of 65.2–65.5%. The coverage depth was approximately ∼60.9X ± 23.6. Analysis of single nucleotide variants (SNVs) revealed an average of approximately 788 ± 111 SNPs, along with approximately 51 ± 8 insertions and 47 ± 7 deletions. Notably, the majority of the InDels were short (≤20 nt, as shown in Table 1). The distribution of the SNPs encompassed approximately 105 ± 18 intergenic, 372 ± 47 non-synonymous, and 232 ± 36 synonymous variations. Genome annotation highlighted the existence of approximately 4294 ± 14 genes, consisting of 4254 coding sequence (CDS) and 44 transfer RNA (tRNA) genes, with functional assignments identified in approximately 3378 ± 12 cases. Approximately 1061 ± 4 proteins were annotated with Enzyme Commission (EC) numbers, whereas approximately 918 ± 4 exhibited Gene Ontology (GO) assignments. Moreover, approximately 814 ± 3 proteins were successfully linked to the KEGG pathways. The assignment of roughly 1961 ± 19 genes was carried out within subsystems, which represent clusters of proteins collaborating to execute specific biological processes or structural complexes (Table S1).

**Figure 1:**
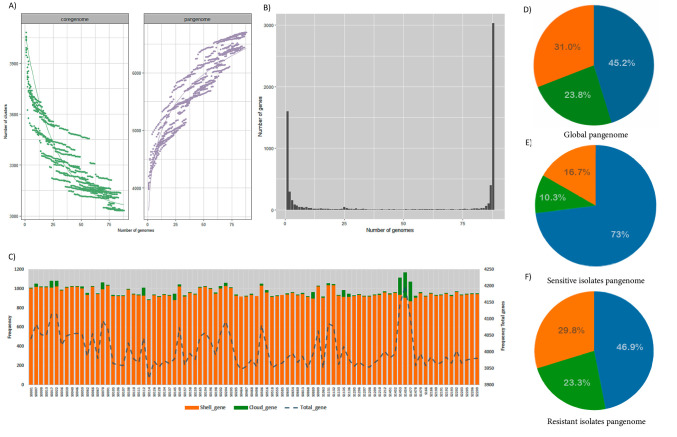
**The pan-genome of the 88 *Mtb* isolates from Ecuador.** (**A**) Rarefaction curves of the core genome (green) and the pangenome (light purple). (**B**) Cluster frequency in the core genome. Each bar represents the core genome composition of an individual isolate, with the global core genome depicted by the final bar after analysis by Roary for all isolates. (**C**) Distribution of accessory, cloud, and total genes across all isolates analyzed in this study, highlighting the variability in pangenome components among different strains. (**D**) Global pangenome composition, where the core genome constitutes almost half of the total gene content. (**E**) Pangenome composition for sensitive isolates, with the core genome comprising nearly 75% of all genes. (**F**) Pangenome composition for resistant isolates, where the accessory genes increase, potentially reflecting specific characteristics associated with resistance. Colors in pies: core gene (blue), accessory gene (orange), and cloud gene (green). The dotted line in panel C represents the total genes of each isolate.

Pangenome analysis of the 88 *Mtb* isolates by Roary revealed a pangenome consisting of 6703 gene families, which comprised 3032 core genes, 2073 accessory genes, and 1598 cloud genes (Figure 1A–D). The ratio between core and pangenome size was 0.45, indicating that the genome comprised almost half of the pangenome (45.2%), which is indicative of the highest variability. Pathway analysis revealed that 19.2% of the annotated protein-coding genes were associated with amino acid metabolism, followed by genes related to carbohydrates (16.2%), lipid metabolism (13.6%), and xenobiotics, biodegradation, and metabolism (12.5%). Genes involved in amino acid biosynthesis were found to be conserved and essential for pathogenicity in bacteria, including *Mtb* [46]. Through the BPGA pipeline, our analysis revealed that the pangenome consisted of an average of 4397 genes. These genes were categorized into a core genome comprising 3104 genes, ∼659 accessory genes, and 767 unique genes. The sensitive isolates had a higher number in the core genome whilst in the resistant isolates, the accessory genome comprised more genes than the sensitive (Figure 1E,F). According to the pangenome evaluation, the “b” value of 0.0861522 in the power-law regression model suggests that the pangenome of *Mtb* is near to close.

Notably, the three *Mtb* isolates with the highest number of cloud genes belonged to the X-type (S1454 [n = 261] and S1477 [n = 200]) and LAM (S1453 [n = 177]) sub-lineages (Figure 1C), and a similar pattern was identified by the BPGA pipeline in these isolates (S1454 [n = 211], S1477 [n = 163]), and S1453 [n = 144]). Only 6.3% (40 of 638) of the cloud genes in these isolates were assigned to COG (Clusters of Orthologous Genes), encompassing lipid transport and metabolism, secondary metabolite biosynthesis, transport and catabolism, amino acid transport and metabolism, and energy production and conversion categories. Notably, 80% of the genes associated with lipid transport and metabolism were found in isolate S1454. In comparison, 75% of the genes associated with energy production and conversion were found in isolate S1477.

A functional analysis among different pangenome partitions based on COG categories globally showed that genes associated with metabolism (45.45% in the core genes to 35.27% in the accessory genes), as well as genes related to cellular processes and signaling (24.97% in the unique gene to 9.79% in the core genes), present significant global variations. Additionally, we determined that genes with general function predictions [R] (over 10% in all partitions) are the most recurrent, followed by genes associated with secondary metabolites biosynthesis, transport, and catabolism [Q] (10% in accessory and unique genes). Finally, genes associated with cell motility [N] were predominantly in the accessory gene category.

Furthermore, according to functional assignments based on the KEGG pathways database, our genomes globally exhibited mostly genes associated with metabolism (core and accessory genes), whereas genes related to environmental information processing were mostly identified as unique genes. Within the core-genome gene distributions, amino acid metabolism (15.0%) had the highest representation, followed by carbohydrate metabolism (14.6%), overview (11.8%), and xenobiotics biodegradation and metabolism (8.9%). Among accessory genes, carbohydrate metabolism (13.9%) and overview (10.0%) were the most prevalent. In the unique gene category, genes associated with the cellular community, digestive system, energy metabolism, immune system, infectious diseases, and signaling molecules and interactions had a similar distribution, each accounting for 8.7%.

### 3.2. Pangenome Diversity on Lineage of Mtb

When we analyzed the core genome composition based on the drug resistance pattern, the sensitive isolate (73.0% by Roary and 83.0% by BPGA) had a more closed pangenome than the resistant isolates (46.8% by Roary and 60.7% by BPGA) (Figure 1E,F). By lineage, the LAM and X-type isolates had a higher number of cloud genes (1073 and 874, respectively); however, by BPGA, the results differed slightly in the core genome, and the largest changes were found mostly in accessory and unique genes (Table 2).

**Figure 2:**
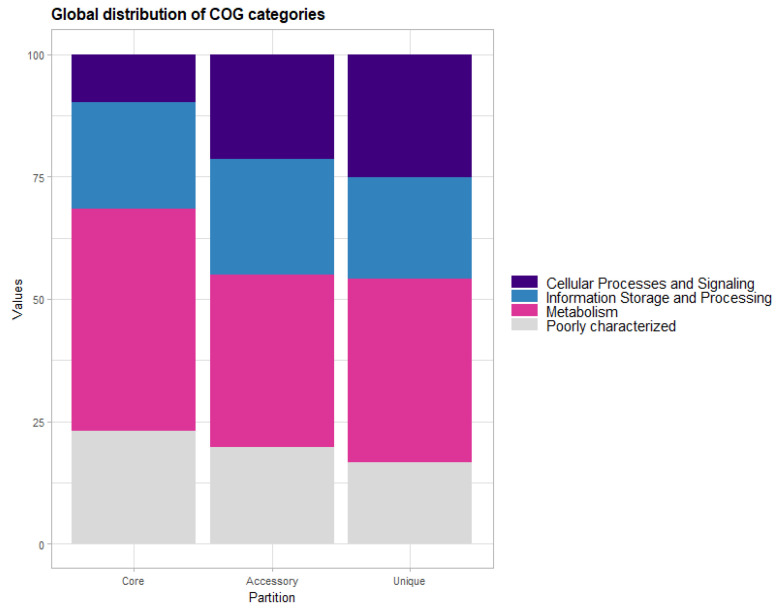
**Distribution of the major COG categories.** Stacked bars represent the percentage distribution of the functional COG annotations among the genes highly conserved in the core, accessory, and unique partitions from all lineages analyzed in this study.

A closer analysis of the distribution within the COG database and KEEG pathways revealed that the diversity in lineage comes mainly from accessory and unique genes. When analyzing the functional annotation of COG major categories for accessory genes, we found that Euro-American isolates had the highest percentage of information storage and processing genes (29.14%), while the S-type lineage had a higher percentage of cellular processes and signaling genes (31.16%), and the X-type lineage exhibited the highest percentages of metabolism and poorly characterized genes (38.29% and 20.0%, respectively). In terms of unique genes, the S-type lineage showed the highest representation of information storage and processing genes (33.24%), followed by Euro-American with cellular processes and signaling genes (30.30%); metabolism had the highest representation in the LAM lineage (48.13%), and the X-type lineage had the highest unique poorly characterized (20.33%). Reviewing the distribution of COG functional categories, we found that genes related to [Q] secondary metabolites biosynthesis, transport, and catabolism genes (found in all six lineages), and [N] cell motility genes (presented predominantly in Haarlem, LAM, mainly-t, S-type, and X-type) were the most prevalent, accounting for more than 10% in each lineage. Additionally, [R] general function prediction genes were exclusively present in the LAM, S-type, and X-type lineages with the highest percentages (Figure 2 and Table S2).

**Figure 3:**
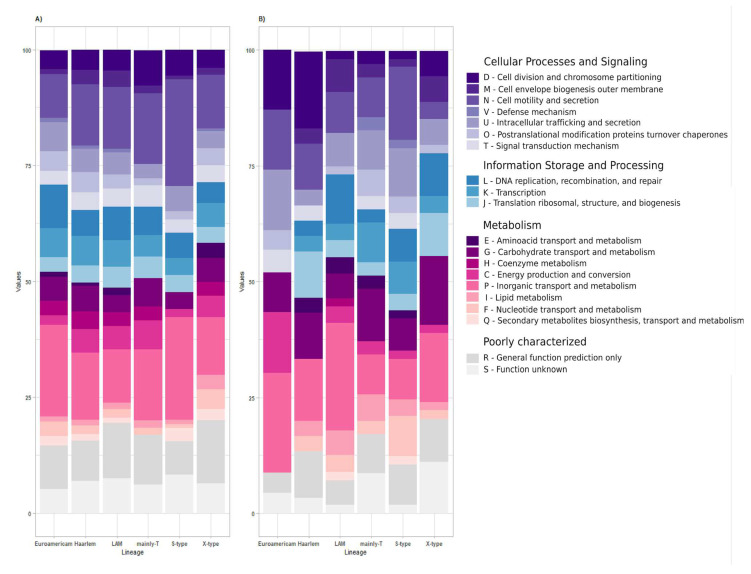
**Distribution of primary COG categories.** Stacked bars represent the percentage distribution of the functional annotations among the genes highly conserved on (**A**) the accessory partition; (**B**) the unique genes from all lineages analyzed in this study. **Functional Annotation Groups.** *Cellular Processes and Signaling*. D: cell division and chromosome partitioning; M: cell envelope biogenesis outer membrane; N: cell motility and secretion; V: defense mechanism; U: intracellular trafficking and secretion; O: posttranslational modifications proteins turnover chaperones; T: signal transduction mechanism. *Information Storage and Processing*. L: DNA replication, recombination, and repair; K: transcription; J: translation ribosomal, structure, and biogenesis. *Metabolism*. E: amino acid transport and metabolism; G: carbohydrate transport and metabolism; H: coenzyme metabolism; C: energy production and conversion; P: inorganic transport and metabolism; I: lipid metabolism; F: nucleotide transport and metabolism; Q: secondary metabolites biosynthesis, transport, and metabolism. *Poorly characterized*. S: function unknown; R: general function prediction only.

Among the unique genes, the Euro-American isolate exhibited greater diversity, primarily composed of genes related to Cell cycle control, cell division, chromosome partitioning, cell motility, intracellular trafficking, secretion, vesicular transport, energy production, and conversion (12.9% each). Additionally, a significant portion of their unique genes were associated with secondary metabolites biosynthesis, transport, and catabolism (21.65%). In contrast, the X-type lineage had a notable representation of genes involved in carbohydrate transport and metabolism, as well as secondary metabolites biosynthesis, transport, and catabolism (14.79% each). Furthermore, a proportion of their unique genes fell into the category of function unknown (11.09%) (Figure 3 and Table S3).

**Figure 4:**
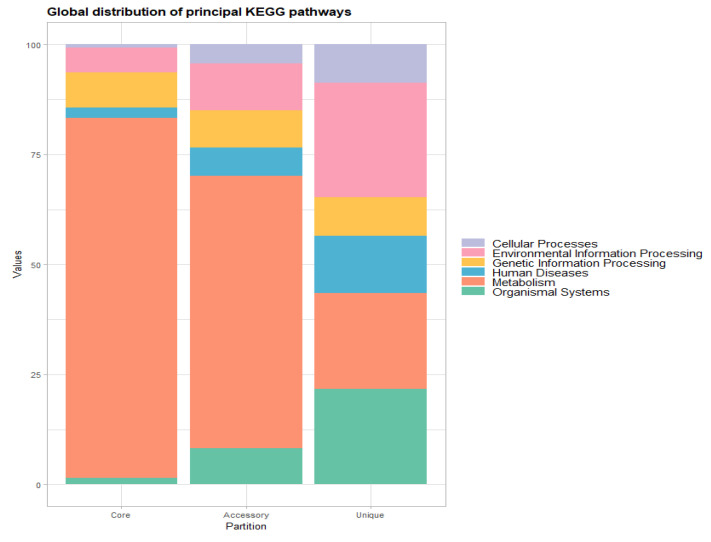
**General distribution of KEGG pathways**. Differences in the percentage of functional KEGG pathways annotated in the core, accessory, and unique partitions from all lineages analyzed in this study.

From Global Metabolic KEEG pathways, the mainly-T isolate significantly contributed to the accessory partition. It had the highest percentage of genes related to environmental information processing (33.3%), followed by organismal systems (25.0%) and cellular processes (12.5%). Metabolism had the highest representation, with percentages of 57.96% in the X-type lineage, 54.14% in LAM, and 53.57% in Haarlem. Human diseases accounted for 13.64% of genes in the S-type lineage, and genetic information processing represented 11.05% in LAM. Regarding unique genes, all lineages exhibited a significant presence of genes related to environmental information processing, human diseases, and organismal systems, followed by cellular processes (Euro-American, Haarlem, LAM, and S-type). Metabolism genes were notably represented in the LAM, mainly-T, and S-type lineages. However, only the X-type lineage showed the highest percentage of genes associated with genetic information processing (15.38%) (Figure 4 and Table S2).

**Figure 5:**
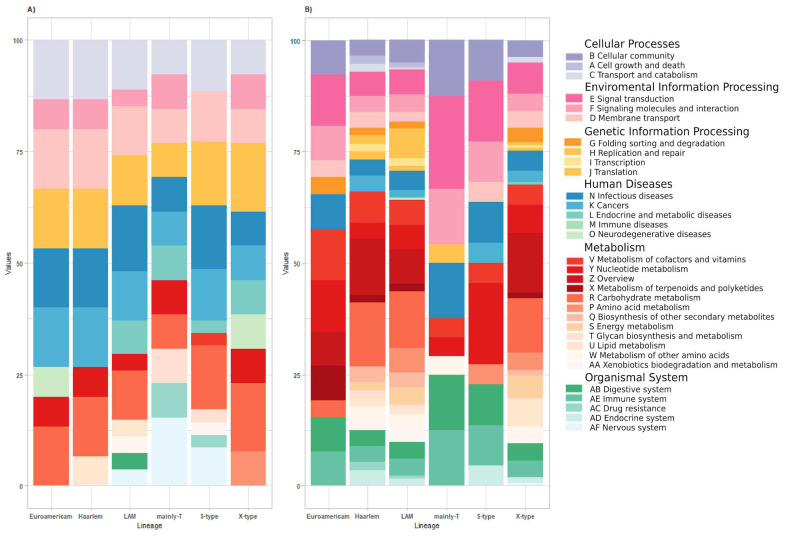
**Distribution of primary KEGG pathways.** (**A**) The percentage of functional annotations among the genes highly conserved among the unique genes from all lineages analyzed; (**B**) the percentage of functional annotations among the genes highly conserved among the accessory genes from all lineages analyzed in this study. Alphabetical labels correspond to standard KEGG pathway categories, with full names included to ensure clarity and consistency with KEGG conventions, facilitating the interpretation of genes according to their biological roles.

However, a more detailed analysis of accessory genes, based on the KEGG pathways distribution, reveals that carbohydrate metabolism genes exhibited a higher level of diversity. The Haarlem lineage represented 14.3% of carbohydrate metabolism genes, followed by LAM (12.7%) and X-type (12.10%). Similarly, signal transduction genes displayed significant variability, with the mainly-T isolate having 20.8%, S-type with 13.6%, and Euro-American with 11.5%. Notably, the mainly-T- and S-type isolates lacked genes associated with carbohydrate metabolism (Figure 5A).

From the unique genes, infectious diseases were prevalent across almost all lineages (except mainly-T) followed by the cellular community, digestive system, immune system, signaling molecules and interaction, and signal transduction. The Euro-American and Haarlem isolates represented 11.3% of these categories, while the LAM lineage had 11.11%, and the S-type lineage had 11.43% (increasing to 14.29% in signal transduction genes). It is worth noting that several pathways in the case of accessory genes did not involve any genes (Figure 5B and Table S4).

### 3.3. Genetic Diversity of the Beta-Lactamase Resistance-Associated Genes

To identify the existence of mutations that may affect genes related to beta-lactamase susceptibility, we analyzed the 88 Ecuadorian *Mtb* genomes to identify the SNPs present in 46 genomic regions commonly identified as beta-lactamase resistance-associated and cell wall biosynthesis genes of MTBC [47]. In all the isolates, 107 SNPs were identified in 46 genomic regions. *murD* (132 SNPs), *ddlA* (86 SNPs), *rpfC* (30 SNPs), *rpfE* (99 SNPs), *ponA1* (95 SNPs), *dacB2* (29 SNPs), *ftsK* (51 SNPs), *Rv0192* (93 SNPs), and *Rv0008c* (104 SNPs) genes showed the highest accumulation of SNPs distributed into various substitutions among the characterized resistance profiles (Table 3 and Table S5). These genes were primarily associated with peptidoglycan biosynthesis. Typically, the MDR isolates presented the highest number of mutations. The lineages that frequently exhibited a higher number of SNPs were LAM and S-type. Six substitutions were present in all isolates: one synonymous (Ala189Ala in *eccA2*) and five non-synonymous (Arg247Gly in *murD*, Thr365Ala in *ddlA*, Arg126Gln in *rpfE*, Ser127Pro in *Rv0192*, and Ser145Pro in *Rv0008c*).

The *ftsk* gene (Rv2748c) displayed the highest number of substitutions, with six non-synonymous and two synonymous substitutions. Among these, *Met298Val* substitution was the most frequent (47.0%, 24/51) and was only identified in the LAM isolates. In *murD* (Rv2155c), among the three nonsynonymous mutations, the most frequent was *Arg247Gly*, predominantly present in MDR isolates (52/88 isolates), with LAM and S-type isolates accounting for 67% of the cases. In contrast, *Phe76Leu* was exclusively present in S-type isolates. The *ponA1* (*Rv0050*) gene exhibited five substitutions, with *Pro631Ser* (43/95) and *Ala244Ala* (33/95) being the most common.

Additionally, with regard to substitutions distributed in all isolates, mutations were identified in different frequencies according to the lineages, as follows: In the LAM isolates more than 76% of them had mutations in genomic regions *pknA*, *mmaA4*, *hisI*, *rpfC*, *cut3*, *papA1*, *ponA1*, *Rv0791c, Rv0948c, Rv1987, Rv2022c, Rv3057c*, and *Rv3365c*. For the Haarlem isolates, 100% presented mutations in the genomic regions *pknA*, *Rv1128c*, *pheT*, *hsdM*, *Rv3057c*, *cut3*, *eccA2*, *murD*, *Rv3915*, and *ponA1* and a deleterious effect mutation in the *eccA*2 gene (Gln460*). Among S-type isolates, mutations in the genomic regions *pheT*, *murD*, *rpfE*, *ftsK*, *ftsH*, and *Rv0008c* were found in more than 90% of the isolates. Finally, in X-type isolates, mutations in *Rv0324*, *Rv1128*, *hsdM*, *cut3*, *eccA2*, *murD*, and *ponA1* were identified in 100% of the isolates, whereas *lpqK*, *glmU*, *rpfB*, and *ponA1* (Thr58Ala) were identified in 76% of the isolates.

## 4. Discussion

Several studies have been conducted to identify the causes of specific clinical behavior in different isolates of *Mtb* [48]. However, different results have been obtained depending on the geographical location [49,50,51]. The present study sought to characterize the genetic composition of Ecuadorian isolates and ascertain whether this pangenome displays variations associated with lineages or specific resistance patterns. This is the first comprehensive study analyzing the pangenome composition in Ecuadorian isolates. At the time of this study, a single investigation was undertaken involving 21 isolates [36], highlighting the necessity for an expanded collection of sequenced isolates from Ecuador.

Pangenome studies provide evidence of gene loss or gain events within a species, which depend on the environment in which they thrive [52]. In the case of *Mtb*, its environment is restricted, resulting in limited diversity [53]. Our analyses using Roary and BPGA reveal significant disparities in pangenome determination. Among the 88 *Mtb* isolates examined, the pangenome derived from the Roary algorithm encompassed 48% more accessory genes and unique genes compared to the results from the BPGA algorithm. Furthermore, the core genome constitutes 45% (Roary) or 70% (BPGA) of the total pangenome. These findings not only diverge from each other but also deviate from previous reports, which describe a core genome accounting for 25% [54], approximately 86% [55], or about 74% [35] of the pangenome. Roary assignment suggests that accessory genes will continue to increase. Different studies attribute variations in the number of annotated and assigned genes within pangenome partitions to the different thresholds used by the algorithms for gene annotation, clustering, and classification [29,34,56,57]. Therefore, it is important to standardize the processes used to define the pangenome and its partitions, particularly for prokaryotes.

Functional analysis showed that, among the lineages identified in our study, gene assignments were predominantly associated with metabolism (~45%) in the core genome, specifically lipid (~7.8%) and amino acid (~7.3%) transporters. In accessory and unique genes, assignments were related to secondary metabolites biosynthesis, catabolism, and transport (~15.9%), particularly in the LAM and S-type lineages. These findings are consistent with other studies where major genes are associated with lipid metabolism [35,55]. In fact, lipid metabolism holds significant relevance for *Mtb*, primarily because of its critical role as a component of the cellular membrane and mass, along with its involvement in vital processes such as cell invasion, evasion of the immune system, virulence, and growth retardation. Moreover, the identification of accessory and unique genes mostly associated with the biosynthesis, catabolism, and transport of secondary metabolites highlights the adaptive capacity of *Mtb* to the host or external environment [48,58,59]. These factors would be associated with differences in transmissibility and virulence characteristics observed in the members of the *Mtb complex*, playing a crucial role in disease recurrence [35,49].

Furthermore, the presence of cloud genes within these accessory and unique gene categories underscores the genetic diversity that contributes to the pathogen’s adaptability. These cloud genes may encode functions that are not essential for survival under standard laboratory conditions but become critical under specific environmental pressures encountered during infection or treatment. For instance, cloud genes could facilitate enhanced metabolic pathways or provide mechanisms for resisting host immune responses or antibiotic action [60]. This genetic variability can lead to significant differences in strain behavior, including variations in virulence and transmission dynamics among different populations of M. tuberculosis. Understanding these cloud genes is essential for developing targeted interventions and improving our strategies for TB control by addressing the unique characteristics of various strains within this complex pathogen.

Studies have revealed that isolates from the LAM lineage (4.3.4.2) exhibit increased susceptibility to beta-lactams, whereas the Beijing and Haarlem lineages (4.1.2.1) display high resistance. Our results identified the highest number of mutations in isolates of LAM and S-type sublineages, suggesting that mutations in cell wall biosynthesis genes could be associated with higher resistance to beta-lactams, which should be further investigated if beta-lactams are considered for inclusion in TB treatment regimens. No mutations in the *blaC* gene were determined. Mutations in resuscitation-promoting factors (*Rpfs*), which are related to PG hydrolases, have been associated with increased outer membrane permeability and susceptibility to beta-lactams [61,62], and a similar pattern has been observed with substitutions in *EccA*2, an ESX-2 type VII secretion system component. Our findings suggest that Ecuadorian isolates related to the Euro-American and Haarlem sublineages may exhibit higher resistance to amoxicillin and meropenem because of the substitution Glu215Gly. Conversely, mutations in *lpqK*, a conserved lipoprotein similar to penicillin-binding proteins (PBPs), and *RpfC* were linked to increased susceptibility in the strains. In our study, we identified the substitutions Glu67Lys and His16Arg in the *lpqK* and *rpfC* genes, respectively, mostly in LAM isolates.

Despite the fact that we identified mutations associated with β-lactamase resistance by analyzing genomic sequences from clinical isolates, the absence of microbiological susceptibility results can be attributed to several limitations. First, most clinical microbiology laboratories do not routinely perform susceptibility testing for beta-lactams in *Mtb*, as these antibiotics are not part of standard TB treatment regimens. Traditional susceptibility testing methods, such as disk diffusion or broth microdilution, may not be effective in detecting beta-lactam resistance in *Mtb* due to its complex cell wall structure. Additionally, other resistance mechanisms, such as alterations in porins or efflux pumps, may confer resistance without being detected by conventional methods. Moreover, pharmacokinetic factors may limit the concentration of beta-lactams at the site of infection, hindering the ability to detect resistance in laboratory assays. Resistance may also not be fully expressed phenotypically under the conditions used for routine testing.

It is important to note that while we have identified lineage-associated signatures and mutations potentially linked to beta-lactam resistance, experimental beta-lactamase data were not included in this study due to the technical limitations mentioned above. As beta-lactams are not routinely tested in *Mtb* for clinical management, microbiological sensitivity testing was not conducted for these drugs in our isolates. Furthermore, existing methodologies in *Mtb* resistance testing often focus on first-line and second-line drugs rather than beta-lactams, which are not part of standard treatment protocols. Given the potential role of beta-lactams in TB treatment, we acknowledge the need for experimental validation of resistance mechanisms and suggest that further studies, including susceptibility testing, are warranted to assess the full susceptibility profile of these antibiotics in *Mtb*.

## Figures and Tables

**Table 1:** Summary of detected genomic variants in the lineages of 88 *Mtb* isolates identified in this study.

Lineages	SNP	Deletions		Insertions	
		More than 20 nt	Less than 20 nt	More than 20 nt	Less than 20 nt
Euro-American	900	55	4	55	4
Haarlem	877	45	3	55	3
LAM	760	43	4	45	2
mainly T	494	33	2	32	1
S-type	760	37	4	47	3
X-type	863	45	6	55	3

SNP: Single nucleotide polymorphism.

**Table 2:** Pangenome composition according to drug resistance and lineage features of 88 *Mtb* isolates from Ecuador.

		Roary Pipeline				BPGA Pipeline			
	n	Core Genes	Accessory Gene	Cloud Gene	Pangenome	Core Genes	Accessory Gene	Cloud Gene	Pangenome
Total	88	3032	2073	1598	6703	3104	659	767	4397
Genomic resistance									
Resistant_isolate	64	3054	1940	1518	6522	3123	646	756	5145
Sensitive_isolate	24	3573	817	504	4894	3477	255	198	4188
Lineages									
LAM	39	3281	1385	1073	5739	3330	443	463	4649
X_Type	21	3306	1281	874	5461	3352	392	563	4655
S_type	10	3684	573	269	4526	3589	150	239	4095
Haarlem	9	3674	593	224	4491	3540	209	190	4146
mainly_T	5	3754	394	290	4438	3618	112	194	3990
Euro-American	4	3760	334	268	4362	3616	144	118	3985

**Table 3:** Principal synonymous and non-synonymous mutations identified in genes related to β-lactamase resistance.

Function	Functional Categories	Locus	Gene Name	AA Change
	Regulatory proteins	*Rv0015c*	*pknA*	Ser385Arg (agc/agG)
	Regulatory proteins	*Rv0324*		Thr168Ala (act/Gct)
	Lipid metabolism	*Rv0642c*	*mmaA4*	Asn165Ser (aac/aGc)
	Conserved hypotheticals	*Rv0791c*	*-*	Ser100Cys (tcc/tGc)
	Intermediary metabolism and respiration	*Rv0948c*	*-*	Lys59Thr (aag/aCg)
	Insertion seqs and phages	*Rv1128c*	*-*	Glu270Gly (gaa/gGa)
	Intermediary metabolism and respiration	*Rv1606*	*hisI*	Thr99Ile (acc/aTc)
	Cell wall and cell processes	*Rv1987*	*-*	Ser36Asn (agt/aAt)
	Conserved hypotheticals	*Rv2022c*	*-*	Val118Ala (gtg/gCg)
	Information pathways	*Rv2756c*	*hsdM*	Leu306Pro (ctg/cCg)
	Intermediary metabolism and respiration	*Rv3057c*	*-*	Asp112Ala (gat/gCt)/His111His (cac/caT)
	Conserved hypotheticals	*Rv3365c*	*-*	Ala266Thr (gcg/Acg)
	Cell wall and cell processes	*Rv3451*	*cut3*	Gly209Asp (ggc/gAc)/Leu259Arg (ctg/cGg)
	Lipid metabolism	*Rv3824c*	*papA1*	Leu35Phe (ctt/Ttt)
	Cell wall and cell processes	*Rv3884c*	*eccA2*	Ala189Ala (gcc/gcG)
PG synthesis	Cell wall and cell processes	*Rv2155c*	*murD*	Arg247Gly (cgg/Ggg)
PG synthesis	Cell wall and cell processes	*Rv2981c*	*ddlA*	Thr365Ala (aca/Gca)
PG hydrolysis	Cell wall and cell processes	*Rv1884c*	*rpfC*	His16Arg (cac/cGc)
PG hydrolysis	Cell wall and cell processes	*Rv2450c*	*rpfE*	Arg126Gln (cgg/cAg)
PG assembly	Cell wall and cell processes	*Rv0050*	*ponA1*	Ala244Ala (gca/gcG)
PG assembly	Conserved hypotheticals	*Rv0192*	*-*	Ser127Pro (tcg/Ccg)
Cell division	Cell wall and cell processes	*Rv0008c*	*-*	Ser145Pro (tcc/Ccc)
Cell division	Cell wall and cell processes	*Rv2748c*	*ftsK*	Met298Val (atg/Gtg)

## Data Availability

Raw reads sequences analyzed in this study can be found in the SRA database under the following accession numbers: PRJNA827129.

## Supplementary Materials

The following supporting information can be downloaded at: https://www.mdpi.com/article/10.3390/biomedicines13020313/s1, Table S1. Genomic and clinical data from the Ecuadorian isolates are shown in Table S2. Distribution of major COG and KEGG pathways from Lineage and pangenome partitions; Table S3. Distribution of primary COG categories from lineage and pangenome partitions; Table S4. Distribution of primary KEGG pathways from lineage and pangenome partitions; Table S5. Synonymous and non-synonymous mutations identified in genes related to ꞵ-lactamase resistance.
