# Supplementary figures and images for: Decoding Ecuadorian Mycobacterium tuberculosis Isolates: Unveiling Lineage-Associated Signatures in Beta-Lactamase Resistance via Pangenome Analysis
Source: Biomedicines. 2025 Jan 28;13(2):313. doi: 10.3390/biomedicines13020313 (PMC11853040; doi:10.3390/biomedicines13020313)

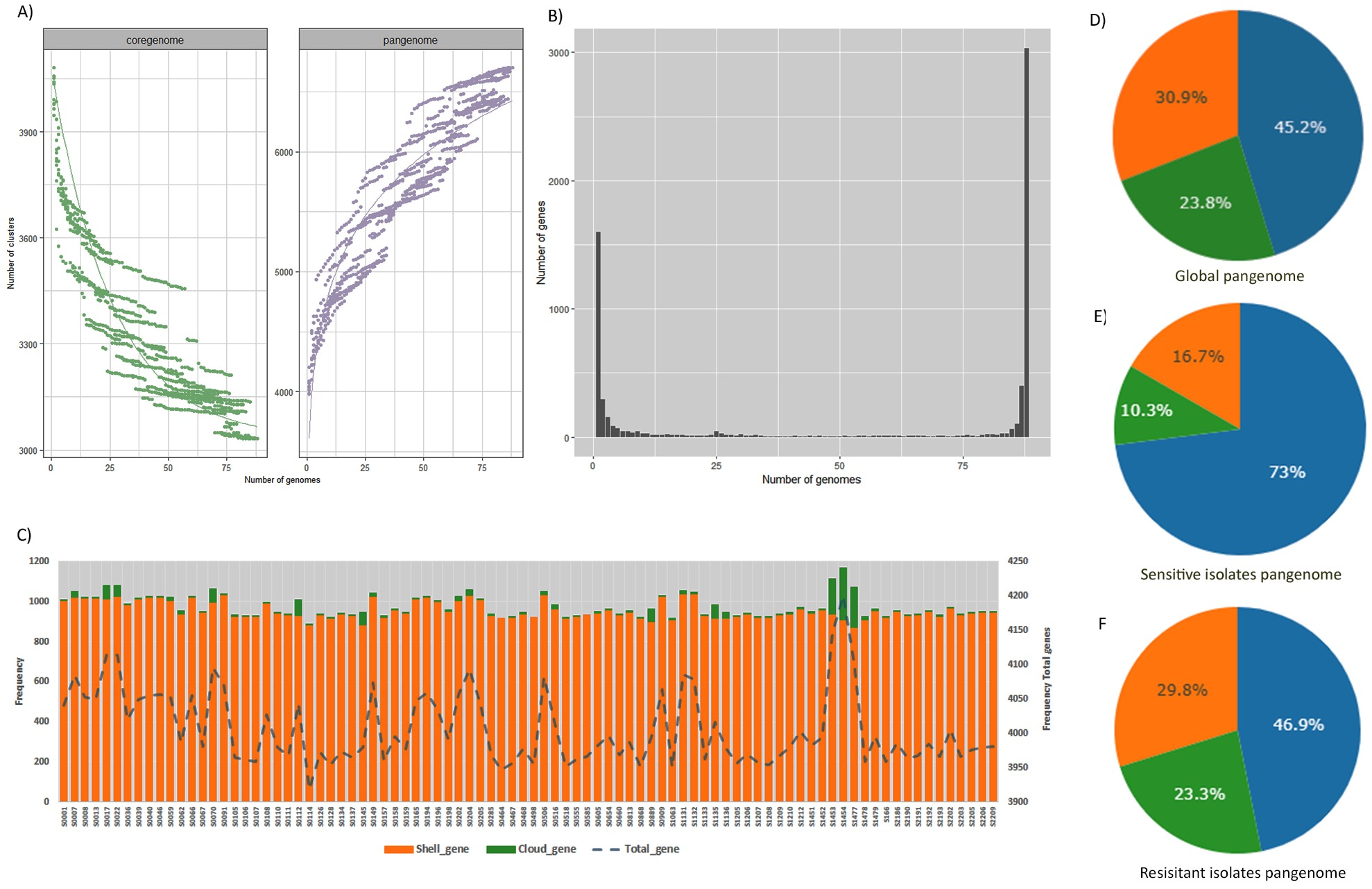

Supplement: Supplementary file 1 [file biomedicines-13-00313-s001.zip › Figure_01_Pangenome_88_samples.tif]

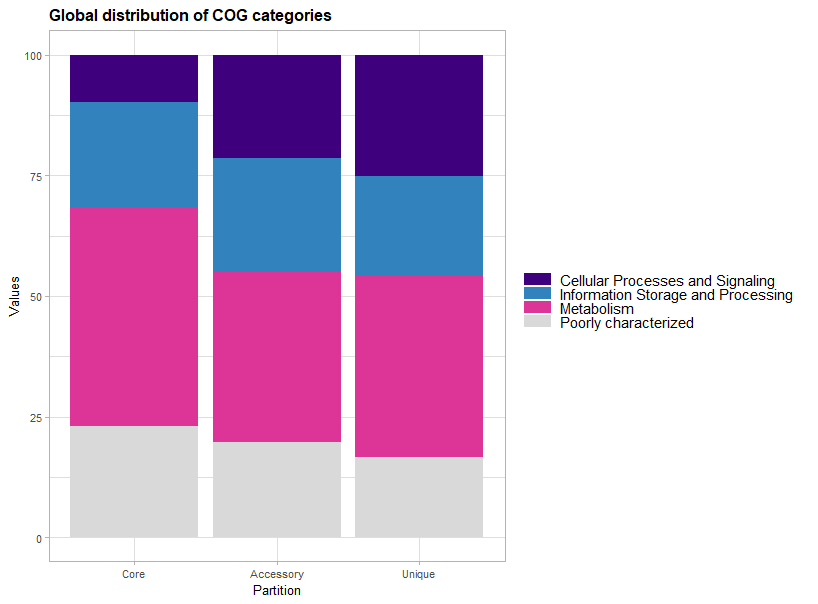

Supplement: Supplementary file 1 [file biomedicines-13-00313-s001.zip › Figure_02_Global_major_COG_distribution.tif]

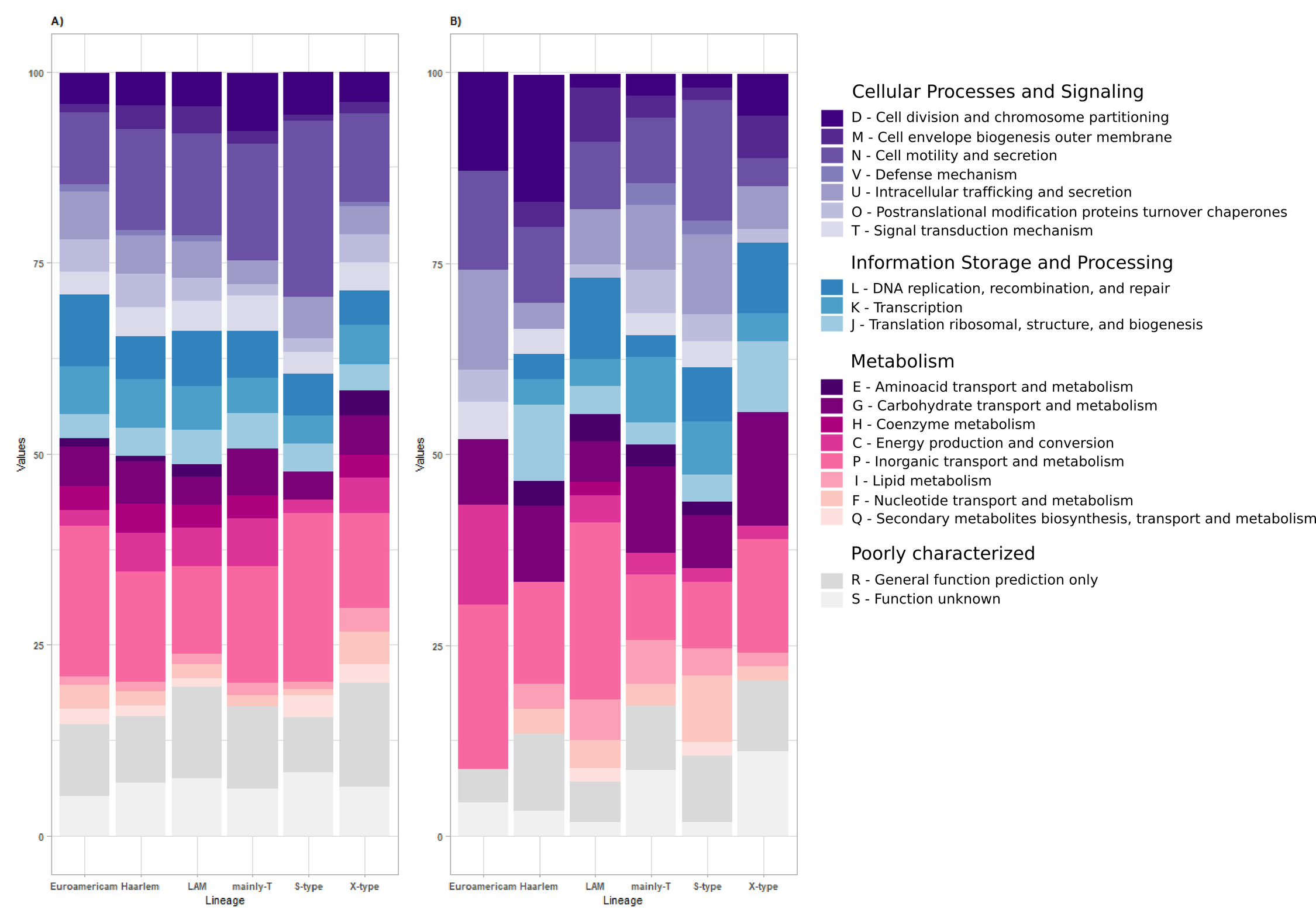

Supplement: Supplementary file 1 [file biomedicines-13-00313-s001.zip › Figure_03_Primary_COG_categories_Accesory_Unique_pangenome.tif]

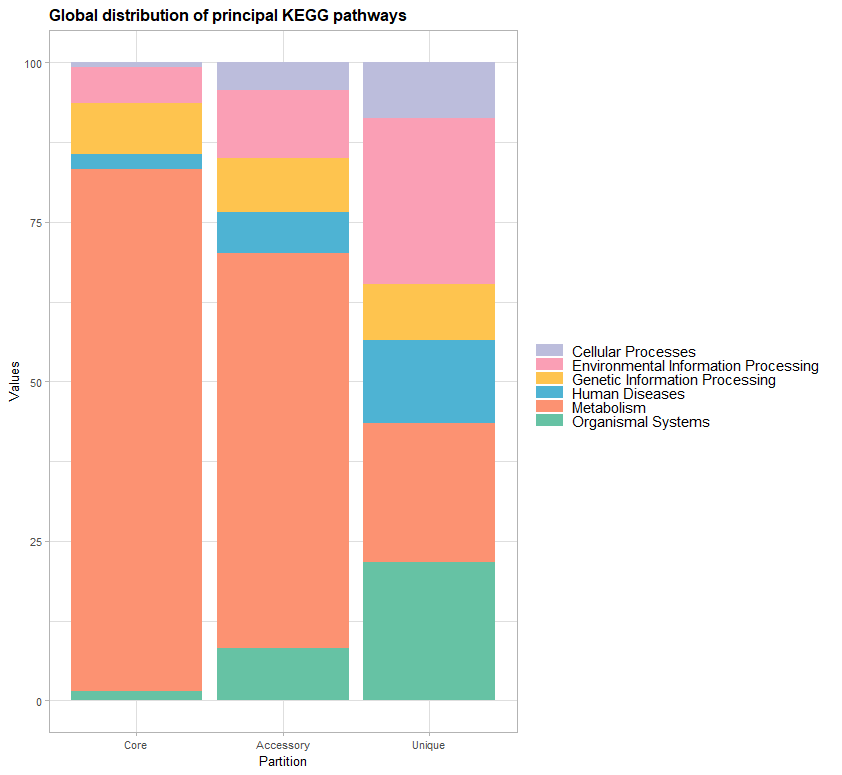

Supplement: Supplementary file 1 [file biomedicines-13-00313-s001.zip › Figure_04_Global_KEGG_pathways.tif]

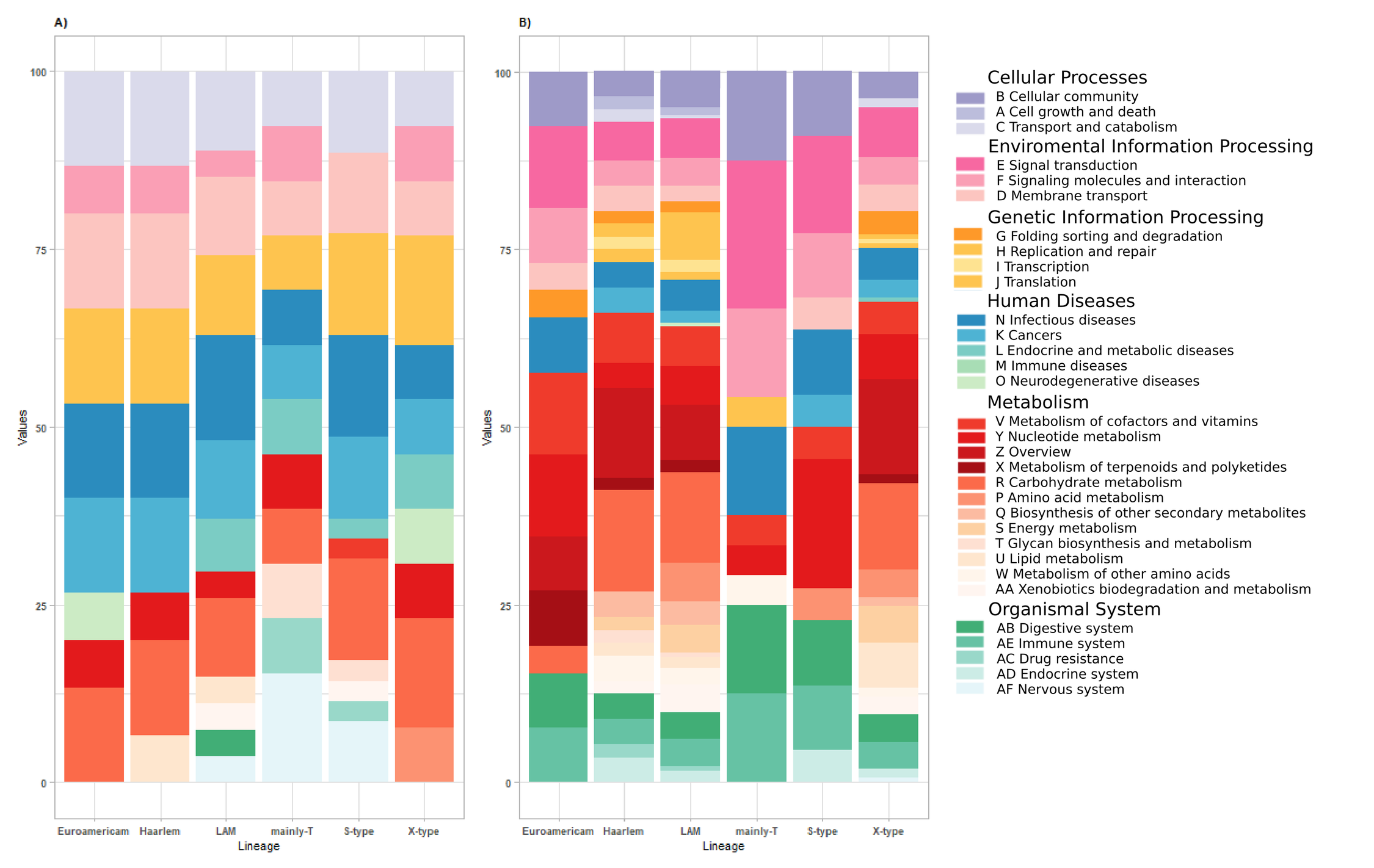

Supplement: Supplementary file 1 [file biomedicines-13-00313-s001.zip › Figure_05_Primary_KEGG_pathways_Accesory_Unique_pangenome.tif]
